# A quantitative analysis of factors which influence supplement use and doping among adolescent athletes in New Zealand

**DOI:** 10.3389/fspor.2023.1069523

**Published:** 2023-02-13

**Authors:** Sian Clancy, Robert Borotkanics, Sarah-Kate Millar, Anthony R. H. Oldham

**Affiliations:** ^1^ Department of Environmental Sciences, Auckland University of Technology, Faculty of Health and Environmental Health Sciences, Auckland, New Zealand; ^2^ Drug Free Sport New Zealand, Auckland, New Zealand

**Keywords:** social norms, volition, autonomy, youth sport, internal perceived locus of control (IPLOC), confidence sourcing

## Abstract

**Objectives:**

Doping is a maladaptive behaviour which poses numerous risks and potentially enhances athletic performance while supplement use poses threats of positive, yet inadvertent, doping control results. Investigation is required to understand factors that influence adolescent supplement use and doping in New Zealand (NZ).

**Design:**

A survey was completed by 660 athletes aged 13 to 18 years, of any gender, who competed at any level of any sport in NZ. Forty-three independent variables measured autonomy, confidence sources, motivational climate, social norms and age.

**Methods:**

Multivariate, ordinal, and binary logistic regression models measured associations between independent variables and five dependant variables: supplement use, doping, doping considerations and intent (soon and in the next year).

**Results:**

Confidence through mastery, internally perceived locus of control (IPLOC) and volition decreased the odds of doping while confidence through self-presentation, subjective and descriptive norms increased the odds of supplement use and doping.

**Conclusion:**

To decrease the odds of doping, adolescent autonomy should be increased in sport through opportunities for volitional decision making and exposure to mastery as a confidence source.

## Introduction

Doping is considered an anti-social and maladaptive behaviour in sport where risks outweigh perceived benefits, particularly during adolescence (1). Prevention is important because doping poses health, reputation, social and financial risks to individuals and sporting organisations (2, 3). More broadly, doping threatens the perceived integrity of sport, challenging the notion that participation develops good character (4). Historically, doping has been assumed specific to elite level athletes as reflected in a body of research targeting high-performing, adult populations. However, evidence of doping has emerged across competitive levels, including among adolescent athletes for whom consequences are pertinent and arguably persistent (5).

During adolescent development, the risks of doping may be amplified by the very nature of its prohibition in sport as athletes may perceive prohibited substance use in sport predominantly in terms of risk (the chance to get caught) and rewards (enhanced performance). This is salient to the adolescent context where perceptions of risk and reward are fluid when making decisions in pursuit of desired outcomes (6). More simply, the relationship between risk and reward may be perceived differently by adolescents. Adolescence is therefore a perilous phase for doping initiation, with longer term concerns as engagement may become habitual and remain in later life (7).

Whether via “shared mental representation” or “gateways”, there appear to be links between supplement use and the likelihood of doping (8, 9). As detailed elsewhere (9), theoretical perspectives of potential “gateways” have suggested that adolescent's supplement consumption may increase doping risks through the development and acceptance of routine substance use to improve sporting performance. The practice of substance use to enhance performance may also initiate cognitive processes associated with doping (8). Exhibiting tenets of the Gateway Theory, supplement use to enhance sporting performance has also been described as an initial step in a sequential trajectory toward doping (9). Notable here, is the risk to athletes of returning inadvertent positive doping control results through the use of supplements which contain prohibited substances (2). In pursuit of interventions that mitigate the potentially life-long impact of doping on adolescents, it is important to understand factors that contribute to a world where supplementation and doping are considered routes to success. Factors that influence these behaviours have not yet been investigated in NZ nor with an adolescent athlete population. Worth considering in this inquiry are variations in influential factors by country, culture and community. Identifying such factors in NZ's sporting context is important given its geographic isolation, its small yet effective athlete population and low occurrence of Anti-Doping rule violations. This permits a more nuanced consideration of factors to guide effective intervention strategies through an investigation of commonly researched factors alongside novel hypotheses. Variables considered here include confidence, basic psychological needs, subjective norms, descriptive norms and motivational climate. These variables have been considered in relation to doping or supplement use, but less commonly in adolescents or within the same dataset.

*Confidence sourcing.* While it has been reported that high school athletes who doped displayed greater confidence than those who did not (10), mechanisms underpinning these outcomes remain unclear. Some insight may be gained from examining confidence sourcing behaviour and literature which posit that athletes draw confidence from multiple sources, the most important being mastery (11). A direct link has already been shown between task involving climates and reduced intentions to dope, in keeping with this confidence sourced through mastery may have a similar effect. Beyond this, contextual variations are possible via self-presentation and preparedness, also dimensions of sport confidence (11). As physical self-perceptions are known to be influenced by supplementation and doping (9), it may be that athletes reliant on physical self-presentation are more inclined to engage in these behaviours. Supplementation and doping may also be seen as an act of preparation in support of confidence (8).

*Basic Psychological Needs.* Athletes who experience self-regulated environments are less likely to dope while those exposed to externally regulated environments are more likely to dope (12). To develop suitable interventions, it would be useful to know whether psychological need satisfaction reduces the odds of doping or supplementation. Of particular interest here is autonomy as a construct of Deci and Ryan's (13) Self-Determination Theory (SDT). Need satisfaction and need thwarting have already been linked to doping intentions (14). More nuanced insights helpful to the development of interventions may however be gained from the Basic Need Satisfaction Scale for Sport (BNSSS) (15). This scale examines three sub-dimensions of autonomous needs: choice, internally perceived locus of control (IPLOC) and volition. Of particular interest are IPLOC and volition which may better predict intrinsic motivation than the provision of choice as they reflect a degree of control and a desire for engagement.

*Motivational climate.* Motivational climates can be mastery-orientated and emphasise improvement, or ego-orientated and compare individual's abilities and prioritise performance (16). Research has shown that ego-orientated motivational climates influence adolescent doping, particularly when winning is promoted at all costs often denying individual's decision-making autonomy (5, 17). These outcomes are unsurprising, whether supplementing and doping share similar constructs remains of interest.

*Social norms.* Evidence has shown that subjective norms orientated against doping have a positive effect on doping avoidance (18). That is, an athlete's perception that people important to them would disapprove if they were to dope has a role in protecting and deterring them from engagement. Conversely, descriptive norms have been recognised to influence adolescent doping intentions (19). The same may not be true of supplementing which may be considered normal, thus not warranting social judgement. Taken together, these variables may be factors influential in the lives of adolescents at risk of doping and germane to developing interventions for prevention.

## Methods

Items in this analysis were drawn from a 77-item, anonymous survey developed as part of a larger study (see supplementary information). Survey items were chosen following a review of previous research on supplement use and doping among adolescent athletes and based on three rationale. First, research techniques deemed effective with adolescent populations in previous doping inquiry were employed (3). Second, as challenges have been articulated to compare evidence across studies due to the wide variation of scales measured (5), items from previous scales were included for comparisons to be made to existing knowledge. Finally, areas were identified that required greater understanding in the current context. Pilot testing informed several minor revisions before content validity was assessed with adolescent educators focussing on congruency with intended outcomes and participant comprehension. The readability of each item was measured using the Flesch-Kincaid reading ease scale, resulting in a score of 73.1 (20). The final survey was considered plain English and deemed satisfactory for adolescent comprehension. The survey was accessible on web enabled devices using Qualtrics software.

Participants recorded their age by year and responded to all survey items in consideration of their main sport. Derived from the Sources of Sport Confidence Questionnaire (SSCQ), a total of 18 items from four original subscales measured demonstration of ability, mastery, environmental comfort and self-presentation (11). In total, twelve items were drawn from the Motivational Climate Scale for Youth Sport (MCSYS, 16). Of these, six items measured mastery-orientated climates and six items measured ego-orientated climates. Social norms were assessed through subjective and descriptive norms using items from previous research (7). Three items measured subjective norms through athlete beliefs that people close and important to them would approve if they doped and three items assessed descriptive norms via athlete perceptions of doping prevalence in their sporting environment (7). Derived from the Basic Needs Satisfaction in Sport Scale (BNSSS), three items measured two aspects of autonomy respectively; IPLOC and volition (15). Items measuring choice were omitted at this point for brevity. Doping and supplement use frequency were measured respectively using a single item from previous research (8). A hypothetical scenario from previous research measured doping consideration (21). Finally, doping intentions were measured using two items from Lazuras and colleagues (7). Throughout the survey, *doping* was labelled *banned drug use* to resolve comprehension issues identified in piloting. Further, definitions and examples of *banned drugs* and *supplements* were given to support participant understanding (1). Institutional ethical approval was then obtained.

The sampling strategy for this survey was purposive as it sought adolescent participants, aged 13 to 18 years of age, who competed in any team or individual sport in NZ, at any level. To ensure participant criteria were met, age selection of *12 or less* (1) or *19 or more* (8) resulted in immediate survey termination. Participants were recruited nationwide, by way of sports organisations and secondary schools. Interested adolescents accessed study information and the survey proper via QR code or electronic link made available by these organisations. Information provided throughout recruitment included reminders of the voluntary, anonymous nature of involvement and an explanation that individual consent would be provided through survey participation. Parental consent was not required due to the anonymous nature of this survey.

Descriptive analyses were performed using standard statistical approaches. Multivariate analyses were conducted using methods applied by Rice and colleagues (22). To summarize, covariates were appraised for multicollinearity using Spearman's rho correlations. Correlations exceeding 0.4 were recorded. Univariate ordinal and logistic regression models were then carried out, and covariates with *p* values of 0.2 or smaller were recorded. Multivariate ordinal and logistic regressions were then conducted for each dependant variable. Collinear variables exceeding 0.4 were not included in the same models, consistent with Graham (23). Model fit was measured for each model using Pearson's Goodness-of-Fit tests for ordinal regressions and Hosmer-Lemeshow tests for logistic regressions. Finally, the Akaike Information Criterion (AIC) was calculated for each model, resulting in rank-ordering whereby the “best approximating model” had the lowest AIC value (24). All data were analysed using SPSS 25.

## Results

Data included in this analysis were drawn from surveys which had been completed in full, from a total cohort of 1,298 participants. Following the removal of data from incomplete surveys, a sample size of six hundred and sixty participants (*n* = 660, sufficiently meeting the requirements of statistical power for this analysis. Descriptive statistics of participants are shown in Table 1.

**Table 1:** Descriptive statistics of participants.

Variable	N	%, Range, Mean, SD, Average
Age (years)	*n* = 660	R = 13–18 years
		M = 15 years
		SD: 1.54
Sport type		
Team	*n* = 401	60.8%
Individual	*n* = 259	39.2%
Weekly engagement in sport	*n* = 660	Average = 9.51 h
Gender		
Female	*n* = 434	65.8%
Male	*n* = 224	33.9%
Gender diverse	*n* = 2	0.3%

Univariate regression results of the effect of covariates on each dependent variable are shown in Supplementary Material. Two independent variables had a statistically significant effect on increased odds of supplement use among adolescent athletes: descriptive norms (*OR *=* *1.61, *95% CI =* 1.37, 1.90, *ρ =* <.001) and subjective norms (OR = 1.48, 95% CI = 1.21, 1.82, *ρ* = <.001), Table 2. No independent variables had a statistically significant effect on decreased odds of supplement use.

**Table 2:** Summary of models that increase the odds of supplement use, doping, doping intentions and doping consideration among adolescent athletes.

Dependent variable	Independant variable					95% Confidence Interval	
		OR	SE*β*	t	*ρ*	Lower Bound	Upper Bound
Supplement Use	Subjective norms	1.48	0.10	14.2	<.001	1.21	1.82
	Descriptive norms	1.61	0.08	33.1	<.001	1.37	1.90
Doping	Subjective norms	4.22	0.25	33.1	<.001	2.58	6.90
	Descriptive norms	2.58	0.24	15.5	<.001	1.61	4.14
Doping intentions: in the next year	Subjective norms	2.61	0.10	77.4	<.001	2.10	3.23
Doping consideration	Confidence via self-presentation	2.35	0.28	8.78	0.003	1.33	4.14
	Subjective norms	6.20	0.22	64.9	<.001	3.98	9.66
Doping intentions: soon	Subjective norms	5.81	0.27	40.9	<.001	3.39	9.98

Two independent variables had a statistically significant effect on increased odds of doping: subjective norms (*OR *= 4.22, *95% CI =* 2.58, 6.90, *ρ =* <.001) and descriptive norms (*OR *= 2.58, *95% CI =* 1.61, 4.14, *ρ =* <.001), Table 2. In contrast, volition had a statistically significant effect on decreased odds of doping (*OR *= 0.43, *95% CI =* 0.29, 0.66, *ρ =* <.001), Table 3.

**Table 3:** Summary of models that that decrease the odds of doping, doping intentions and doping consideration among adolescent athletes.*.

Dependent variable	Independant variable					95% Confidence Interval	
		OR	SEβ	t	ρ	Lower Bound	Upper Bound
Doping	Volition	0.43	0.21	15.1	<.001	0.29	0.66
Doping intentions: in the next year	Volition	0.68	0.08	20.1	<.001	0.57	0.80
Doping consideration	Confidence via mastery	0.55	0.24	5.82	0.016	0.34	0.89
Doping intentions: soon	IPLOC	0.53	0.35	3.19	0.074	0.26	1.06
	Volition	0.46	0.25	9.25	0.002	0.28	0.76

* No models measured factors that decreased the odds of supplement use due to a lack of influence identified in earlier regressions.

Subjective norms had a statistically significant effect on increased odds that participants would intend to dope in the next year (*OR* = 2.61, *95% CI* = 2.10, 3.23, *ρ* = <.001), Table 2. Conversely, volition had a statistically significant effect on decreased odds of intentions to dope in the next year (*OR* = 0.68, *95% CI* = 0.57, 0.80, *ρ* = <.001), Table 3.

Two independent variables had a statistically significant effect on increased odds of doping consideration: subjective norms (*OR *= 6.20, *95% CI =* 3.98, 9.66, *ρ =* <.001) and confidence through self-presentation (*OR *= 2.35, *95% CI =* 1.33, 4.14, *ρ =* 0.003), Table 2. Conversely, confidence through mastery had a statistically significant effect on decreased odds of doping consideration (*OR *= 0.55, *95% CI =* 0.34, 0.89, *ρ =* 0.016), Table 3.

Subjective norms had a statistically significant effect on increased odds of intentions to dope soon (*OR* = 5.81, *95% CI* = 3.39, 9.98, *ρ* = <.001), Table 2. Two factors had a statistically significant effect on decreased odds that participants would intend to dope soon: volition (*OR* = 0.46, *95% CI* = 0.28, 0.76, *ρ* = 0.002) and IPLOC (*OR* = 0.53, *95% CI* = 0.26, 1.06, *ρ* = 0.074), Table 3.

## Discussion

Results identify a novel relationship between confidence sourcing and doping. Volition and IPLOC were also found to influence the likelihood of doping, adding nuance to existing results. Interestingly, subjective and descriptive norms were the only factors influencing adolescent supplement use.

Confidence sourced through mastery decreased the odds of doping consideration, intent, and engagement during adolescence, while confidence via self-presentation increased the odds of doping consideration (Table 2). Associations identified between confidence sources and doping in this study support existing knowledge about the influence of adolescent perspectives on self-presentation on increased odds of supplement use and doping (25). These findings are consistent with developmental literature identifying associations between adolescent egocentrism, appearance obsession and substance use to change self-presentation (26). Confidence sourcing through mastery is also considered salient across genders and sport types (11). Given the likely role of coaches, parents, and broader social forces in the development of confidence, it seems important to focus on contextual as well as personal factors in the prevention of doping.

Volition significantly decreased the odds of doping and doping intentions; it also influenced the likelihood of doping consideration (Table 2). In contrast to feeling forced to do things they don't want to do, volition reflects one's desire, willingness and opportunity to implement actions required to perform a specific task (27). Researchers have described doping as a deliberate action (28) however these findings indicate that adolescents do not perceive a need, nor want, to dope to meet the demands of their sport. Similarly, IPLOC decreased doping intentions (Table 2). IPLOC is an individuals' perception of pursuing, initiating and regulating their sporting goals and behaviours (15). Together, these results imply a need to support the development of personally derived anti-doping motives in addition to providing choice. It should be noted however that this cannot be stated unequivocally in the absence of a direct comparison with perceived behaviour control.

Descriptive norms had a statistically significant effect on increased odds of adolescent doping and supplement use while also influential to increased odds of doping consideration and intent (in the next year). Previous research similarly identified descriptive norms as a significant predictor of adolescent doping intentions (19). Consistent with these findings, adolescents have been argued to perceive higher descriptive norms of doping than the suggested 1-5% prevalence (5). Given the influence of norms on increased odds of doping and supplementation, there exists a need to directly challenge adolescent perceptions of doping acceptance and prevalence.

Subjective norms were the only independent variable to exhibit statistically significant, increased odds across all dependant variables (Table 1). Consistent with past research, the influence of subjective norms on behaviour appear salient during adolescence (29). Developmental literature similarly highlights the susceptibility of adolescents to the influence and intensity of subjective norms during this stage which coincides with hyper-awareness of others' opinions and social acceptance (26). These outcomes reinforce the need for subjective norms to be considered a priority in the prevention of adolescent doping. It was surprising that *anti-doping* subjective norms did not influence decreased odds of doping, doping consideration or intentions in these findings. This absence of influence contrasts existing evidence which identified that athlete perceptions of others' disapproval was influential to doping avoidance (18). Furthermore, subjective norms that reject doping have had a protective influence against doping and have promoted anti-doping orientations among adolescents (17). This is consistent with developmental literature which stated that adolescents characteristically avoid the behaviours they anticipate may receive social disapproval (26). The absence of evidence to suggest that subjective norms had an anti-doping influence among adolescents warrants further investigation. Finally, it should be noted that subjective and descriptive norms were the only variables to effect the odds of supplement use. Whether seen through a “Gateway” or “shared representation” lens, these results highlight the normalisation of supplementation as a potential antecedent to doping after which other interpersonal forces might come to bear.

## Conclusion

This research has identified factors that decrease adolescent's odds of doping, doping consideration, and intent. To reduce these odds, attention should be given to increasing athlete's exposure to mastery as a confidence source, reducing focus on appearance and supporting volition/IPLOC as a part of sport development. Results inform practical implications to prevent doping among adolescent athletes in NZ. A contrasting lack of evidence regarding factors that decrease the likelihood of adolescent supplement use warrants further research.

Several practical implications emerged from this investigation which warrant attention from practitioners and organisers of adolescent sport:

• To reduce the odds of doping, opportunities for mastery need to be emphasised in adolescent sport. In this environment, common narratives between appearance and performance should also be challenged.
• Practices that increase volition and empower adolescents to make choices of personal relevance should be promoted in youth sport. Support should also be provided for adolescents to develop and discuss individual reasons for avoiding doping.
• In adolescent sporting environments, focus should be placed on transparently rejecting doping and emphasising related behaviours as undesirable. Adolescent perceptions of doping among their peers should also be challenged. To do so, athletes and support personnel should become familiar with evidence on adolescent doping prevalence, which is reported to be lower than assumed, both nationally (30) and internationally (5).
• Subjective and descriptive norms appear to influence supplementation, which has been argued to lead to doping. All those involved in adolescent sport need to understand and weigh the multifaceted risks associated with supplementing against potential performance benefits.

A limitation of this study includes its cross-sectional nature which renders associations, rather than causality, all that could be drawn. A further limitation of this study is the exclusion of ‘choice’ as a third measure of the SDT's autonomy construct. As IPLOC and volition, both aspects of autonomy within Deci and Ryan's SDT (13), decreased the odds of adolescent doping (volition) and doping intent (volition and IPLOC), future research may benefit from exploring the effect of all aspects of adolescent autonomy on this behaviour. In addition to identifying factors which increase the odds of supplementation and doping, future research should investigate factors which influence the avoidance of doping during adolescence. Further, analysis of a gendered difference was not implemented here as this study sought to identify factors of influence for a wide adolescent community. A gendered examination of this evidence remains relevant however and an area for future research. The outcomes of such advances in knowledge from varied perspectives would benefit future interventions by focusing on factors which influence adolescent decision making about engagement in, and avoidance of, supplement use and doping.

## Data Availability

The datasets presented in this study can be found in online repositories. The names of the repository/repositories and accession number(s) can be found below; http://hdl.handle.net/10292/13768.
