# Supplementary material for: A quantitative analysis of factors which influence supplement use and doping among adolescent athletes in New Zealand
Source: Front Sports Act Living. 2023 Feb 13;5:1069523. doi: 10.3389/fspor.2023.1069523 (PMC9968871; doi:10.3389/fspor.2023.1069523)
Supplement: Supplementary file 1 [file Table1.docx]

Supplementary Information: Survey

| SCALE | ITEM | RESPONSE OPTIONS |
| --- | --- | --- |
| Age | How old are you today (years)? | 12 or less (1), 13 (2), 14 (3), 15 (4), 16 (5), 17 (6), 18 (7), 19 or more (8) |
| IPLOC | In my sport, I feel I am pursuing goals that are my own | Strongly disagree (1), Disagree (2), Neutral (3), Agree (4), Strongly agree (5) |
|  | I feel I participate in sport willingly | Strongly disagree (1), Disagree (2), Neutral (3), Agree (4), Strongly agree (5) |
|  | In my sport, I really have a sense of wanting to be there | Strongly disagree (1), Disagree (2), Neutral (3), Agree (4), Strongly agree (5) |
| Volition | In my sport, I feel I am doing what I want to be doing | Strongly disagree (1), Disagree (2), Neutral (3), Agree (4), Strongly agree (5) |
|  | In my sport, I feel that I am being forced to do things I don’t want to do (reversed scored item) | Strongly disagree (1), Disagree (2), Neutral (3), Agree (4), Strongly agree (5) |
|  | I choose to participate in my sport according to my own free will | Strongly disagree (1), Disagree (2), Neutral (3), Agree (4), Strongly agree (5) |
| Confidence source: Demonstrating ability | I usually gain self-confidence in my sport…when I win | Strongly disagree (1), Disagree (2), Neutral (3), Agree (4), Strongly agree (5) |
|  | when I demonstrate I am better than others | Strongly disagree (1), Disagree (2), Neutral (3), Agree (4), Strongly agree (5) |
|  | when I show my ability by winning | Strongly disagree (1), Disagree (2), Neutral (3), Agree (4), Strongly agree (5) |
|  | when I know I can outperform others | Strongly disagree (1), Disagree (2), Neutral (3), Agree (4), Strongly agree (5) |
|  | when I prove I am better than opponents | Strongly disagree (1), Disagree (2), Neutral (3), Agree (4), Strongly agree (5) |
|  | when I show I am one of the best | Strongly disagree (1), Disagree (2), Neutral (3), Agree (4), Strongly agree (5) |
| Confidence source: Mastery | when I master a new skill | Strongly disagree (1), Disagree (2), Neutral (3), Agree (4), Strongly agree (5) |
|  | when I improve my performance on a skill | Strongly disagree (1), Disagree (2), Neutral (3), Agree (4), Strongly agree (5) |
|  | when I improve my skills | Strongly disagree (1), Disagree (2), Neutral (3), Agree (4), Strongly agree (5) |
|  | when I increase the number of skills I can perform | Strongly disagree (1), Disagree (2), Neutral (3), Agree (4), Strongly agree (5) |
|  | when I develop new skills and improve | Strongly disagree (1), Disagree (2), Neutral (3), Agree (4), Strongly agree (5) |
| Confidence source: Self-presentation | when I feel good about my weight | Strongly disagree (1), Disagree (2), Neutral (3), Agree (4), Strongly agree (5) |
|  | when I feel I look good | Strongly disagree (1), Disagree (2), Neutral (3), Agree (4), Strongly agree (5) |
|  | when I feel my body looks good | Strongly disagree (1), Disagree (2), Neutral (3), Agree (4), Strongly agree (5) |
| Confidence source: Environmental comfort | when I follow certain rituals (e.g. when I do the same things before each competition) | Strongly disagree (1), Disagree (2), Neutral (3), Agree (4), Strongly agree (5) |
|  | when I perform in an environment I like | Strongly disagree (1), Disagree (2), Neutral (3), Agree (4), Strongly agree (5) |
|  | when I feel comfortable in the environment where I am performing | Strongly disagree (1), Disagree (2), Neutral (3), Agree (4), Strongly agree (5) |
|  | when I like the environment I am performing in | Strongly disagree (1), Disagree (2), Neutral (3), Agree (4), Strongly agree (5) |
| Motivational climate: Ego | Winning is the most important thing for the coach | Strongly disagree (1), Disagree (2), Neutral (3), Agree (4), Strongly agree (5) |
|  | The coach spends less time with the athletes who aren’t as good | Strongly disagree (1), Disagree (2), Neutral (3), Agree (4), Strongly agree (5) |
|  | The coach tells us which athletes on the team are the best | Strongly disagree (1), Disagree (2), Neutral (3), Agree (4), Strongly agree (5) |
|  | The coach pays most attention to the best athletes | Strongly disagree (1), Disagree (2), Neutral (3), Agree (4), Strongly agree (5) |
|  | Athletes are taken out of games if they make a mistake | Strongly disagree (1), Disagree (2), Neutral (3), Agree (4), Strongly agree (5) |
|  | The coach tells us to try to be better than our teammates | Strongly disagree (1), Disagree (2), Neutral (3), Agree (4), Strongly agree (5) |
| Motivational climate: Mastery | The coach says that all of us are important to the team’s success | Strongly disagree (1), Disagree (2), Neutral (3), Agree (4), Strongly agree (5) |
|  | The coach says that teammates should help each other improve their skills | Strongly disagree (1), Disagree (2), Neutral (3), Agree (4), Strongly agree (5) |
|  | The coach tells us that trying our best is the most important thing | Strongly disagree (1), Disagree (2), Neutral (3), Agree (4), Strongly agree (5) |
|  | The coach makes athletes feel good when they improve at a skill | Strongly disagree (1), Disagree (2), Neutral (3), Agree (4), Strongly agree (5) |
|  | The coach encourages us to learn new skills | Strongly disagree (1), Disagree (2), Neutral (3), Agree (4), Strongly agree (5) |
|  | The coach tells athletes to help each other get better | Strongly disagree (1), Disagree (2), Neutral (3), Agree (4), Strongly agree (5) |
| Social norms: Subjective norms | Most people I know would think it was OK if I used banned drugs to improve my performance | Strongly disagree (1), Disagree (2), Neutral (3), Agree (4), Strongly agree (5) |
|  | Most people who are important to me would want me to use banned drugs to improve my performance | Strongly disagree (1), Disagree (2), Neutral (3), Agree (4), Strongly agree (5) |
|  | Most people who are close to me would like me to use banned drugs to improve my performance | Strongly disagree (1), Disagree (2), Neutral (3), Agree (4), Strongly agree (5) |
| Social norms: Descriptive norms | At your level of competition in NZ, what percentage of athletes do you think use banned drugs to improve their performance? | (Percentage scale) |
|  | In your sport, how often do you know of banned drug use? | Never (1), Rarely (2), Sometimes (3), Often (4), Always (5) |
|  | How many athletes do you know who use, or have used, banned drugs to improve their performance? | None (1), A few (2), Some (3), Several (4), A lot (5) |
| Supplement Use | How frequently do you use supplements to improve your performance? | Never (1), Rarely (2), Sometimes (3), Often (4), Very frequently (5) |
| Doping | How frequently do you use banned drugs to improve your performance? | Never (1), Rarely (2), Sometimes (3), Often (4), Very frequently (5) |
| Doping Consideration | If, under medical supervision, a banned drug was offered to you which was free or cheap, was not detectable and could make a useful difference to your performance, how much consideration would you give to taking it? | None (1), A little (2), Some (3), More (4), A lot (5) |
| Doping Intentions | Do you think you will use banned drugs to improve your performance soon? | No (1), Yes (2) |
|  | In the next year, how likely are you to use banned drugs to improve your performance? | Not likely (1), A little (2), Likely (3), Quite (4), Very likely (5) |
