# Supplementary material for: A quantitative analysis of factors which influence supplement use and doping among adolescent athletes in New Zealand
Source: Front Sports Act Living. 2023 Feb 13;5:1069523. doi: 10.3389/fspor.2023.1069523 (PMC9968871; doi:10.3389/fspor.2023.1069523)
Supplement: Supplementary file 2 [file Datasheet1.pdf]

Supplementary material 1: Univariate regression results of the effect of covariates on the odds of supplement use

Independent Variable	OR	SEβ	t	ρ	95% Confidence Interval	
					Lower Bound	Upper Bound
IPLOC 1	1.30	0.15	3.25	<.001	1.06	1.59
IPLOC 2	1.01	-0.05	-0.92	.354	0.82	1.24
IPLOC 3	1.24	0.06	1.09	.275	1.00	1.54
Volition 1	1.19	-0.01	-0.31	.753	0.95	1.50
Volition 2*	0.89	-0.02	-0.66	.509	0.76	1.05
Volition 3	1.01	-0.05	-1.03	.300	0.82	1.24
Subjective norm 1	1.67	0.10	2.29	.022	1.36	2.04
Subjective norm 2	1.60	0.03	0.65	.510	1.22	2.10
Subjective norm 3	1.65	0.00	0.00	.996	1.28	2.14
Descriptive norm 1	1.00	-0.00	-0.10	.914	1.00	1.01
Descriptive norm 2	1.71	0.13	3.23	<.001	1.46	2.01
Descriptive norm 3	1.65	0.07	1.59	.112	1.38	1.96
SSC: Demonstration ability 1	1.45	0.07	1.49	.135	1.22	1.72
SSC: Demonstration ability 2	1.40	0.06	1.06	.288	1.22	1.60
SSC: Demonstration ability 3	1.36	-0.05	-0.86	.388	1.17	1.59
SSC: Demonstration ability 4	1.47	0.07	1.14	.254	1.29	1.69
SSC: Demonstration ability 5	1.38	0.01	0.28	.777	1.21	1.59
SSC: Demonstration ability 6	1.36	-0.01	-0.20	.837	1.19	1.56
SSC: Mastery 1	0.99	-0.05	-0.93	.352	0.82	1.19
SSC: Mastery 2	1.01	-0.02	-0.38	.699	0.82	1.23
SSC: Mastery 3	1.03	0.03	0.49	.623	0.83	1.29
SSC: Mastery 4	0.98	-0.03	-0.66	.504	0.80	1.20
SSC: Mastery 5	1.07	0.04	0.70	.481	0.85	1.34
SSC: Self-presentation 1	1.20	-0.01	-0.31	.755	1.05	1.37
SSC: Self-presentation 2	1.25	0.11	1.85	.064	1.09	1.42
SSC: Self-presentation 3	1.20	-0.09	-1.51	.130	1.06	1.37
SSC: Environmental comfort 1	0.98	0.00	0.15	.877	0.82	1.18

SSC: Environmental comfort 2	1.28	0.08	2.04	.042	1.11	1.48
SSC: Environmental comfort 3	1.14	0.04	0.87	.383	0.93	1.36
SSC: Environmental comfort 4	0.97	-0.11	-1.94	.053	0.81	1.16
MC: Ego 1	1.28	0.02	0.49	.624	1.08	1.41
MC: Ego 2	1.15	0.00	0.08	.929	1.01	1.33
MC: Ego 3	1.28	0.04	1.01	.308	1.12	1.46
MC: Ego 4	1.27	0.04	0.79	.428	1.11	1.46
MC: Ego 5	1.14	-0.03	-0.74	.458	1.00	1.30
MC: Ego 6	1.34	0.06	1.45	.145	1.19	1.51
MC: Mastery 1	1.04	0.01	0.29	.765	0.86	1.27
MC: Mastery 2	0.99	0.04	0.91	.358	0.81	1.19
MC: Mastery 3	0.92	-0.02	-0.48	.626	0.78	1.08
MC: Mastery 4	0.94	0.05	1.30	.191	0.81	1.10
MC: Mastery 5	0.90	-0.10	-1.87	.061	0.76	1.06
MC: Mastery 6	1.00	0.04	0.93	.350	0.85	1.18
Age	1.35	0.16	4.28	<.001	1.23	1.48

*Reversed scored item, Internal perceived locus of causality (IPLOC), Sources of sport confidence (SSC), Motivational climate (MC).

Supplementary material 2: Univariate regression results of the effect of covariates on the odds of doping

Independent Variable	OR	SEβ	t	ρ	95% Confidence Interval	
					Lower Bound	Upper Bound
IPLOC 1	0.47	-0.00	-0.04	.967	0.26	0.86
IPLOC 2	0.46	-0.04	-0.81	.415	0.27	0.76
IPLOC 3	0.72	0.11	2.14	.033	0.38	1.35
Volition 1	0.48	-0.10	-2.14	.032	0.28	0.80
Volition 2*	0.44	-0.07	-1.94	.052	0.29	0.66
Volition 3	0.86	0.09	2.26	.024	0.45	1.65
Subjective norm 1	5.30	0.21	5.09	<.001	3.37	8.34
Subjective norm 2	4.28	0.16	3.88	<.001	2.80	6.55
Subjective norm 3	4.86	0.15	3.13	.002	3.17	7.47
Descriptive norm 1	1.04	0.01	0.38	.698	1.02	1.06
Descriptive norm 2	3.71	0.10	2.60	.009	2.42	5.68
Descriptive norm 3	3.55	0.12	3.08	.002	2.47	5.10
SSC: Demonstration ability 1	0.68	-0.04	-0.93	.351	0.40	1.13
SSC: Demonstration ability 2	1.05	0.00	0.00	.998	0.66	1.67
SSC: Demonstration ability 3	0.88	-0.03	-0.24	.804	0.53	1.45
SSC: Demonstration ability 4	1.21	0.02	0.48	.627	0.76	1.93
SSC: Demonstration ability 5	0.99	0.02	0.51	.608	0.62	1.59
SSC: Demonstration ability 6	0.97	-0.05	-1.06	.289	0.61	1.55
SSC: Mastery 1	0.64	0.01	0.28	.780	0.36	1.13
SSC: Mastery 2	0.62	0.03	0.54	.589	0.34	1.13
SSC: Mastery 3	0.48	-0.07	-1.39	.164	0.27	0.84
SSC: Mastery 4	0.59	0.02	0.39	.695	0.32	1.06
SSC: Mastery 5	0.61	0.10	1.94	.053	0.32	1.17
SSC: Self-presentation 1	1.63	0.06	1.33	.182	0.94	2.81
SSC: Self-presentation 2	1.51	0.01	0.17	.863	0.91	2.51
SSC: Self-presentation 3	1.41	-0.02	-0.37	.710	0.88	2.24
SSC: Environmental comfort 1	0.66	-0.01	-0.40	.688	0.38	1.16

SSC: Environmental comfort 2	1.27	-0.01	-0.45	.648	0.76	2.10
SSC: Environmental comfort 3	0.93	-0.04	-0.79	.424	0.51	1.69
SSC: Environmental comfort 4	0.76	0.02	0.39	.690	0.42	1.37
MC: Ego 1	1.59	-0.04	-1.16	.243	1.01	2.51
MC: Ego 2	1.45	-0.06	-1.42	.154	0.92	2.27
MC: Ego 3	2.26	0.02	0.52	.597	1.45	3.51
MC: Ego 4	1.98	0.03	0.75	.453	1.27	3.10
MC: Ego 5	2.15	0.01	0.39	.695	1.40	3.30
MC: Ego 6	1.68	0.02	0.69	.489	1.11	2.52
MC: Mastery 1	0.66	0.03	0.87	.383	0.36	1.22
MC: Mastery 2	0.59	-0.06	-1.32	.185	0.34	1.00
MC: Mastery 3	0.82	-0.05	-1.17	.240	0.48	1.40
MC: Mastery 4	0.82	-0.00	-0.11	.908	0.50	1.37
MC: Mastery 5	1.09	0.06	1.25	.209	0.60	1.97
MC: Mastery 6	0.96	0.08	2.03	.043	0.54	1.71
Age	1.21	-0.02	-0.83	.404	0.87	1.67

*Reversed scored item, Internal perceived locus of causality (IPLOC), Sources of sport confidence (SSC), Motivational climate (MC).

Supplementary material 3: Univariate regression of the effect of covariates on the odds of doping intentions in the next year

Independent Variable	OR	SEβ	t	ρ	95% Confidence Interval	
					Lower Bound	Upper Bound
IPLOC 1	0.75	0.01	0.24	.806	0.61	0.93
IPLOC 2	0.70	0.01	0.30	.761	0.56	0.87
IPLOC 3	0.70	-0.11	-2.13	.033	0.56	0.87
Volition 1	0.93	0.15	2.84	.005	0.73	1.18
Volition 2*	0.68	-0.08	-2.12	.034	0.57	0.80
Volition 3	0.72	-0.05	-1.07	.282	0.58	0.89
Subjective norm 1	2.61	0.24	5.30	<.001	2.10	3.23
Subjective norm 2	2.22	0.10	2.27	.023	1.68	2.93
Subjective norm 3	2.49	0.02	0.51	.609	1.90	3.26
Descriptive norm 1	1.00	0.03	0.90	.365	1.00	1.01
Descriptive norm 2	1.37	0.01	0.46	.643	1.17	1.62
Descriptive norm 3	1.58	0.03	0.79	.427	1.32	1.89
SSC: Demonstration ability 1	1.45	0.08	1.77	.077	1.20	1.74
SSC: Demonstration ability 2	1.54	0.15	2.64	.008	1.32	1.78
SSC: Demonstration ability 3	1.34	-0.01	-0.31	.751	1.14	1.58
SSC: Demonstration ability 4	1.49	0.09	1.59	.111	1.29	1.72
SSC: Demonstration ability 5	1.28	-0.12	-2.13	.033	1.11	1.48
SSC: Demonstration ability 6	1.34	0.01	0.20	.837	1.15	1.55
SSC: Mastery 1	0.81	-0.01	-0.22	.819	0.66	0.98
SSC: Mastery 2	0.79	0.05	0.92	.355	0.68	0.97
SSC: Mastery 3	0.69	-0.08	-1.39	.163	0.55	0.86
SSC: Mastery 4	0.69	-0.11	-2.08	.037	0.56	0.86
SSC: Mastery 5	0.75	0.06	1.03	.300	0.59	0.95
SSC: Self-presentation 1	1.14	-0.00	-0.12	.901	0.99	1.32
SSC: Self-presentation 2	1.29	0.09	1.55	.120	1.12	1.49
SSC: Self-presentation 3	1.27	-0.03	-0.64	.517	1.11	1.45
SSC: Environmental comfort 1	0.79	-0.01	-0.38	.700	0.65	0.95

SSC: Environmental comfort 2	1.04	0.01	0.25	.796	0.90	1.20
SSC: Environmental comfort 3	0.94	-0.06	-1.26	.205	0.79	1.13
SSC: Environmental comfort 4	0.94	0.06	1.07	.281	0.78	1.14
MC: Ego 1	1.23	-0.01	-0.33	.736	1.07	1.42
MC: Ego 2	1.24	-0.01	-0.21	.832	1.07	1.43
MC: Ego 3	1.44	0.10	2.13	.033	1.25	1.65
MC: Ego 4	1.32	-0.01	-0.18	.855	1.15	1.52
MC: Ego 5	1.19	-0.07	-1.72	.085	1.04	1.37
MC: Ego 6	1.18	-0.03	-0.70	.479	1.04	1.34
MC: Mastery 1	0.76	-0.02	-0.51	.606	0.62	0.93
MC: Mastery 2	0.74	-0.00	-0.18	.854	0.60	0.90
MC: Mastery 3	0.82	-0.02	-0.37	.711	0.69	0.83
MC: Mastery 4	0.79	-0.02	-0.51	.604	0.67	0.93
MC: Mastery 5	0.85	0.01	0.28	.777	0.71	1.01
MC: Mastery 6	0.82	0.07	1.58	.115	0.69	0.98
Age	1.19	0.09	2.43	.015	1.08	1.32

*Reversed scored item, Internal perceived locus of causality (IPLOC), Sources of sport confidence (SSC), Motivational climate (MC).

Supplementary material 4: Univariate regression results of the effect of covariates on odds of doping consideration

Independent Variable	OR	SEβ	t	ρ	95% Confidence Interval	
					Lower Bound	Upper Bound
IPLOC 1	0.56	-0.03	-0.78	.431	0.34	0.93
IPLOC 2	0.63	-0.03	-0.75	.449	0.39	1.01
IPLOC 3	0.90	0.10	2.17	.030	0.51	1.59
Volition 1	0.71	-0.02	-0.58	.562	0.42	1.20
Volition 2*	0.64	-0.04	-1.23	.219	0.44	0.93
Volition 3	0.89	0.06	1.64	.101	0.52	1.52
Subjective norm 1	4.82	0.23	5.90	<.001	3.30	7.03
Subjective norm 2	4.15	0.14	3.50	<.001	2.81	6.11
Subjective norm 3	5.30	0.26	5.79	<.001	3.56	7.91
Descriptive norm 1	1.04	0.08	2.47	.014	1.02	1.05
Descriptive norm 2	3.21	0.09	2.53	.012	2.27	4.54
Descriptive norm 3	2.92	0.06	1.72	.085	2.16	3.95
SSC: Demonstration ability 1	0.98	0.01	0.41	.680	0.62	1.57
SSC: Demonstration ability 2	1.09	0.06	1.38	.168	0.75	1.59
SSC: Demonstration ability 3	0.82	-0.10	-2.08	.038	0.55	1.22
SSC: Demonstration ability 4	1.05	-0.02	-0.44	.659	0.73	1.51
SSC: Demonstration ability 5	1.05	0.09	1.75	.080	0.72	1.54
SSC: Demonstration ability 6	0.91	-0.07	-1.62	.105	0.63	1.33
SSC: Mastery 1	0.67	-0.05	-1.05	.292	0.42	1.07
SSC: Mastery 2	0.74	0.11	2.24	.025	0.44	1.25
SSC: Mastery 3	0.55	-0.01	-0.25	.802	0.34	0.89
SSC: Mastery 4	0.59	-0.02	-0.58	.556	0.36	0.96
SSC: Mastery 5	0.71	0.09	1.89	.059	0.40	1.25
SSC: Self-presentation 1	1.59	0.08	1.89	.059	1.03	2.46
SSC: Self-presentation 2	1.29	-0.06	-1.28	.201	0.87	1.90
SSC: Self-presentation 3	1.43	0.02	0.40	.688	0.98	2.08
SSC: Environmental comfort 1	0.60	-0.05	-1.42	.154	0.38	0.93

SSC: Environmental comfort 2	1.47	0.04	1.18	.237	0.96	2.25
SSC: Environmental comfort 3	0.82	-0.10	-2.23	.026	0.51	1.31
SSC: Environmental comfort 4	0.76	0.05	1.10	.269	0.47	1.22
MC: Ego 1	1.41	-0.02	-0.66	.509	0.98	2.05
MC: Ego 2	1.07	-0.04	-1.08	.279	0.73	1.57
MC: Ego 3	1.52	0.04	1.11	.265	1.08	2.15
MC: Ego 4	1.21	-0.01	-0.37	.705	0.84	1.74
MC: Ego 5	1.26	0.00	0.07	.941	0.89	1.80
MC: Ego 6	1.40	-0.00	-0.02	.984	1.01	1.95
MC: Mastery 1	0.83	0.01	0.32	.748	0.49	1.41
MC: Mastery 2	0.85	-0.03	-0.72	.467	0.51	1.43
MC: Mastery 3	0.95	-0.08	-1.76	.077	0.60	1.50
MC: Mastery 4	0.86	-0.01	-0.29	.768	0.57	1.31
MC: Mastery 5	1.69	0.10	2.27	.023	0.97	2.92
MC: Mastery 6	0.95	0.08	2.31	.021	0.60	1.52
Age	1.17	-0.04	-1.32	.187	0.90	1.51

*Reversed scored item, Internal perceived locus of causality (IPLOC), Sources of sport confidence (SSC), Motivational climate (MC).

Supplementary material 5: Univariate regression results of the effect of covariates on the odds of intent to dope soon

Independent Variable	OR	SEβ	t	ρ	95% Confidence Interval	
					Lower Bound	Upper Bound
IPLOC 1	0.41	-0.04	-1.13	.258	0.21	0.78
IPLOC 2	0.50	-0.04	-0.90	.364	0.28	0.89
IPLOC 3	0.81	0.11	2.26	.024	0.39	1.71
Volition 1	0.51	-0.04	-0.83	.406	0.28	0.92
Volition 2*	0.42	-0.07	-2.00	.046	0.27	0.67
Volition 3	0.92	0.08	2.02	.043	0.43	1.97
Subjective norm 1	3.65	0.20	4.85	<.001	1.59	8.38
Subjective norm 2	1.90	0.20	4.70	<.001	0.70	5.14
Subjective norm 3	1.43	0.22	4.77	<.001	0.60	3.38
Descriptive norm 1	1.00	0.01	0.46	.640	0.97	1.04
Descriptive norm 2	1.99	0.04	1.25	.209	0.92	4.28
Descriptive norm 3	0.68	0.03	0.78	.432	0.29	1.59
SSC: Demonstration ability 1	1.48	0.04	0.93	.350	0.61	3.54
SSC: Demonstration ability 2	1.78	0.06	1.28	.201	0.75	4.24
SSC: Demonstration ability 3	0.55	-0.03	-0.74	.456	0.21	1.44
SSC: Demonstration ability 4	1.09	-0.04	-0.69	.486	0.46	2.58
SSC: Demonstration ability 5	1.05	0.00	0.09	.928	0.44	2.49
SSC: Demonstration ability 6	0.73	-0.04	-0.88	.379	0.33	1.63
SSC: Mastery 1	0.71	-0.09	-1.81	.070	0.37	1.36
SSC: Mastery 2	1.17	0.15	2.82	.005	0.50	2.72
SSC: Mastery 3	1.27	0.06	1.05	.291	0.38	4.28
SSC: Mastery 4	0.69	0.00	0.05	.956	0.25	1.94
SSC: Mastery 5	0.82	0.01	0.29	.771	0.22	3.06
SSC: Self-presentation 1	1.06	0.04	0.83	.404	0.51	2.22
SSC: Self-presentation 2	0.52	-0.14	-2.48	.013	0.22	1.19
SSC: Self-presentation 3	2.38	0.10	1.96	.050	1.09	5.17
SSC: Environmental comfort 1	0.83	-0.03	-0.76	.448	0.40	1.72

SSC: Environmental comfort 2	1.37	0.01	0.28	.778	0.73	2.57
SSC: Environmental comfort 3	1.13	-0.04	-0.93	.349	0.43	2.95
SSC: Environmental comfort 4	0.51	0.01	0.20	.838	0.18	1.40
MC: Ego 1	1.18	-0.01	-0.34	.731	0.70	1.99
MC: Ego 2	0.86	-0.02	-0.68	.496	0.47	1.56
MC: Ego 3	1.08	-0.00	-0.20	.836	0.57	2.07
MC: Ego 4	1.62	0.07	1.42	.153	0.76	3.47
MC: Ego 5	1.29	-0.00	-0.07	.944	0.74	2.25
MC: Ego 6	1.65	0.03	0.85	.393	0.93	2.93
MC: Mastery 1	1.03	0.03	0.72	.470	0.40	2.67
MC: Mastery 2	0.44	-0.04	-0.93	.348	0.18	1.06
MC: Mastery 3	1.05	-0.01	-0.24	.804	0.40	2.77
MC: Mastery 4	1.07	0.00	0.18	.855	0.52	2.19
MC: Mastery 5	1.76	0.02	0.51	.608	0.66	4.68
MC: Mastery 6	0.98	0.07	1.83	.068	0.43	2.21
Age	1.25	-0.01	-0.41	.679	0.87	1.80

*Reversed scored item, Internal perceived locus of causality (IPLOC), Sources of sport confidence (SSC), Motivational climate (MC).
